# A Rationally Designed TNF-α Epitope-Scaffold Immunogen Induces Sustained Antibody Response and Alleviates Collagen-Induced Arthritis in Mice

**DOI:** 10.1371/journal.pone.0163080

**Published:** 2016-09-22

**Authors:** Li Zhang, Jin Wang, Aizhang Xu, Conghao Zhong, Wuguang Lu, Li Deng, Rongxiu Li

**Affiliations:** 1 State Key Laboratory of Microbial Metabolism, School of Life Sciences & Biotechnology, Shanghai Jiao Tong University, Shanghai 200240, China; 2 Institute of Medical Science, Department of Pharmacology and Physiology, School of Medicine, Shanghai Jiao Tong University, Shanghai 200025, China; 3 Laboratory of Cellular and Molecular Biology, Jiangsu Province Institute of Traditional Chinese Medicine, Nanjing, 210028, Jiangsu, China; 4 Engineering Research Center of Cell & Therapeutic Antibody, Ministry of Education; Shanghai 200240, China; 5 Shanghai HyCharm Inc., 1 Fute Street East, Bldg. 458, Rm. 518, Shanghai 200131, China; Wayne State University, UNITED STATES

## Abstract

The TNF-α biological inhibitors have significantly improved the clinical outcomes of many autoimmune diseases, in particular rheumatoid arthritis. However, the practical uses are limited due to high costs and the risk of anti-drug antibody responses. Attempts to develop anti-TNF-α vaccines have generated encouraging data in animal models, however, data from clinical trials have not met expectations. In present study, we designed a TNF-α epitope-scaffold immunogen DTNF7 using the transmembrane domain of diphtheria toxin, named DTT as a scaffold. Molecular dynamics simulation shows that the grafted TNF-α epitope is entirely surface-exposed and presented in a native-like conformation while the rigid helical structure of DTT is minimally perturbed, thereby rendering the immunogen highly stable. Immunization of mice with alum formulated DTNF7 induced humoral responses against native TNF-α, and the antibody titer was sustained for more than 6 months, which supports a role of the universal CD4 T cell epitopes of DTT in breaking self-immune tolerance. In a mouse model of rheumatoid arthritis, DTNF7-alum vaccination markedly delayed the onset of collagen-induced arthritis, and reduced incidence as well as clinical score. DTT is presumed safe as an epitope carrier because a catalytic inactive mutant of diphtheria toxin, CRM197 has good clinical safety records as an active vaccine component. Taken all together, we show that DTT-based epitope vaccine is a promising strategy for prevention and treatment of autoimmune diseases.

## Introduction

TNF-α is a pleiotropic pro-inflammatory cytokine playing pivotal roles in both physiological and pathological processes [1]. The primary function of TNF-α is to regulate immune cells in inflammation as well as in protective immune responses against a variety of infectious pathogens. As a master regulator of pro-inflammatory cytokines such as IL-1β, IL-6 and GM-CSF [2], over expression of TNF-α causes a variety of chronic inflammatory diseases including rheumatoid arthritis, Crohn’s disease, psoriatic arthritis, ankylosing spondylitis and psoriasis, and so on [3].

Blockade of TNF-α activities with monoclonal antibodies (e.g. Infliximab, Adalimumab) as well as a receptor-immunoglobulin fusion protein (e.g. Etanercept) [3–5] significantly improved clinical outcomes, in particular, rheumatoid arthritis. Nevertheless, all TNF-α biological inhibitors that have been approved for clinical uses are limited in practice due to both high manufacture costs and the risk of anti-drug antibodies (ADAs) response [6, 7]. It is therefore imperative to develop novel strategies to circumvent these shortcomings.

Active immunization against TNF-α has been intensively investigated as an alternative approach to address these limits [8]. So far, TNF-α vaccines based on the whole molecule such as TNF-K and TNF AutoVaccIne have shown remarkable results in animal studies [9, 10]. However, these vaccine strategies are not successful in the human trials. TNF-K is a conjugate mixture of TNF-α and KLH. The bioactivity of the cytokine is inactivated by formaldehyde treatment. TNF AutoVaccIne comprises two recombinant TNF-α proteins with specific peptides replaced by a CD4 T cell epitope from tetanus toxin. In both cases, the elicited antibody responses against the endogenous molecule were weak while humoral responses against the immunogens or partially denatured TNF-α were strong, suggesting that the epitopes are compromised during production processes [11, 12]. Thus a rational design is necessary to generate a stable structure feasible to manufacture.

Since TNF-α is a potent cytokine, a whole molecule immunogen need inactivated either by chemical modification such as formaldehyde treatment [10] or site directed mutagenesis [13]. However, both approaches unavoidably compromise the immunogenicity of the immunogen. Therefore, a peptide epitope based vaccine design is a preferred choice. Work from Capini *et al* have shown that a cyclic TNF-α epitope peptide (aa 80–96) conjugated to KLH elicits a stronger neutralizing antibody response than the linear counterpart, which suggests that stabilizing the conformation of the peptide epitope is a key criterion in the vaccine design [14]. Here, we developed an epitope-scaffold immunogen against TNF-α, in which the conformation of the epitope peptide TNF-α aa 80–97 is stabilized by transplantation onto a scaffold molecule, a transmembrane domain of diphtheria toxin (DTT). We assessed the immunogenicity of the vaccine against native TNF-α in mice as well as the therapeutic efficacy in collagen-induced arthritis mouse model. Our results showed that DTT-based epitope-scaffold vaccine is a promising strategy for prevention and treatment of autoimmune diseases.

## Materials and Methods

### Mice

For immunization, we used 54 BALB/c mice (females, aged 6–7weeks), 6 mice per experimental group. For vaccine efficacy in CIA mouse model, we used 19 DBA/1J mice (males, aged 6 weeks), 9 mice in the DTNF7 group, and 10 mice in the DTT control group. All mice were purchased from SLAC Laboratory Animal Centre (Shanghai, China) and kept in specific pathogen-free conditions in Shanghai Jiao Tong University Animal Center. All animal studies were performed in accordance with institutional guidelines and with approval by the Institutional Animal Care and Use Committee of Shanghai Jiao Tong University. Euthanasia was carried out using CO_2_ according to American Veterinary Medical Association Guidelines. The mice were inspected on a daily-basis for signs of ill health and distress. We euthanized the animals at the end of the experiments since there was no mice that reached humane endpoints including weight loss by 20% or more, severe lameness, dyspnea, ruffled fur, weakness, dehydration, or a hunched appearance, blistering at the injection site [15].

### Calculation of TD and DD

Various number of amino acid residues at position 89–96 on diphtheria toxin T-domain (DTT, aa 202–378 of DT) corresponding to aa 290–297 of DT were replaced by the TNF-α epitope using Discovery Studio 3.5. The resulting epitope-scaffold proteins denoted as DTNF proteins were named DTNF1 to DTNF7. In DTNF1 to 6, amino acid residues corresponding to DTT aa 95–96, aa 94–96, aa 93–96, aa 92–96, aa 91–96, aa 90–96 were replaced with TNF-α epitope VSRFAISYQEKVNLLSA (aa 80–96), respectively. In DTNF7, aa 89–96 were replaced with TNF-α epitope VSRFAISYQEKVNLLSAV (aa 80–97). The inter residue distances (TD and DD) were calculated in Swiss-Pdb Viewer 4.0.1 based on the X-ray crystal structures of mouse TNF-α and Diphtheria Toxin (PDB entry 2tnf, 1.4 Å and 1MDT, 2.4Å). TD (Å) stands for the interatomic distance between Valine 80 to Serine 96 or Valine 97 of TNF-α. DD (Å) stands for the interatomic distance between end residues of the replaced segment. The interatomic distance is calculated using the coordinates of the nitrogen atom of the alpha-amino group in N-terminal end residue and the carbon atom of the alpha-carboxy group in C-terminal end residue.

### Protein expression and purification

The proteins were expressed and purified to homogeneity according to Hatem A. Elshabrawy [16]. The DNA encoding DTNF with PreScission protease cleavage site was generated by overlapping PCR, and digested with BamHI and XhoI, cloned into pGEX6P-1. The vector DNA was transformed into BL21 cells. Protein expression was induced with 1mM IPTG (isopropyl-D-thiogalactopyranoside) when the culture reached OD = 0.6. After culturing for additional 12 h, the cells were pelleted by centrifugation, resuspended in PBS, sonicated and the debris was removed by centrifugation. The supernatant was applied to GST-column (GE Healthcare) according to manufacturer’s instruction. GST tag was cleaved with PreScission protease overnight at 4°C, and removed by GST affinity column. The resulting DTNF proteins were further purified through Superdex G75 chromatography.

### Immunogenicity of DTNF proteins

50 μg of purified DTT or DTNF proteins mixed with aluminum hydroxide adjuvant (InvivoGen) were injected into Female BALB/c mice (6–7 weeks old) intradermally followed by three boost injections with 30 μg, 20 μg, 20 μg proteins plus aluminum hydroxide adjuvant on day14, 28, 42. Blood samples were draw by retro-orbital sinus puncture after anesthesia by isoflurane inhalation on day 49. Anti-TNF-α and Anti-DTT Ab titer and IgG subclasses were measured by ELISA.

### Differential Scanning Calorimetry (DSC)

0.5 mg/ml DTNF proteins were degassed and the protein unfolding events were recorded on a VP-Capillary differential scanning calorimeter (MicroCal, LLC) between 35 and 100°C at a scan rate of 1°C /min and under a constant pressure of 30 psi. The data were analyzed using Origin according to manufacturer’s instruction.

### CD spectroscopy

The CD spectra (195–250 nm) were recorded on JASCO J-815 in 1-mm quartz cell at 25°C. Protein concentrations were 0.230 mg/ml.

### Homology modeling and molecular dynamics

The tertiary structure of DTNF proteins were modeled in Modeller9V7 based on the X-ray crystal structure of DT and the mouse TNF-α mentioned above, and validated by Procheck [17]. MD simulations were performed as previously described using Desmond 3.0 in default settings and lasted for 300 ns [18]. The protein structures were visualized using pyMOL 0.99 software.

### Protection of collagen-induced arthritis

CIA was induced and the arthritis score were assessed according to Wei Tang [19]. Briefly, 2 mg/mL chicken type II collagen (Chondrex) mixed with an equal volume of CFA (4mg/mL Mycobacterium tuberculosis H37Ra) in a tissue homogenizer. On day 0, six-week-old DBA/1J mice were anesthetized by isoflurane inhalation according to IACUC, and injected intradermally with 100 μl of the resulting emulsion at the base of the tail. On day 14, the experimental group mice were injected intradermally with 50 μg DTNF7 formulated with alum, and the control group mice were injected with alum and DTT. On day 28 and 42, the mice were boosted with 25 μg immunogen formulated with alum. Mice were monitored on a daily basis for arthritis incidence and evaluated two or three times per week for severity. Each paw was scored using a 0 to 4 scoring system. The paw scores were summed to yield individual mouse scores, with a maximum score of 16 for each mouse. Score 0, normal; 1, mild swelling confined to the tarsal bones or ankle joint; 2, mild swelling extending from the ankle to the tarsal bones; 3, moderate swelling extending from the ankle to the metatarsal joints; and 4, severe swelling encompassing the ankle, foot and digits, or ankylosis of the limb. For histologic analysis by hematoxylin-eosin staining and Safranin O staining, mice were sacrificed on day 56, and hind paws were used.

### Cytokine assays

Sera were diluted 4-fold and the concentration of cytokines (IL-1β, IL-6, IFN-γ, IL-10, IL-17 and TNF-α) were measured using Luminex multiplex technology (Bio-Rad Laboratories) according to manufacturer’s instruction.

### Statistical analysis

The means ± SEM or means ± SE were calculated in Prism 6.0 (GraphPad) from three to ten replicates. Statistical comparisons were performed using t-test, where *P ≤ 0.05,**P ≤ 0.01, or ***P ≤ 0.001 was considered significant.

## Results

### The molecular design of a TNF-α epitope-scaffold immunogen

**Fig 1:**
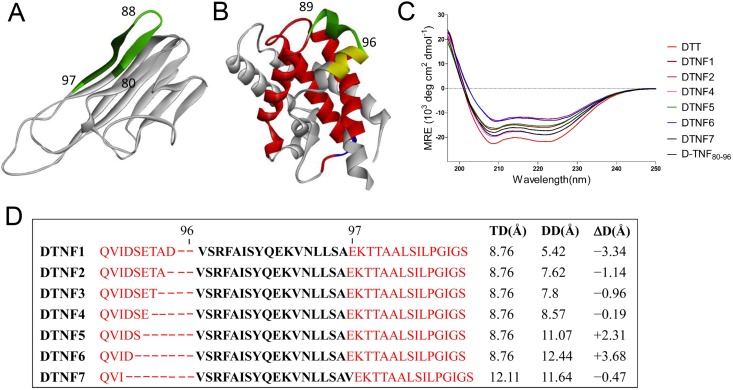
The design of epitope-scaffold DTNF proteins. (A) The crystal structure of the mTNF-α from PDB 2tnf. The neutralizing epitope-containing peptide was green colored. (B) The crystal structure of DTT from PDB 1MDT. The transplantation site is highlighted in green. The position of Th epitopes is indicated in red. The position of conformational perturbation in DTNF2, 5, 6 is colored in yellow, and that in DTNF4 is colored in blue. (C) The circular dichroism spectra of DTT and DTNF proteins. (D) Generation of epitope-scaffold DTNF proteins. Two to eight amino acid residues of DTT at indicated positions (red hyphens) were replaced by the TNF-α epitope peptide (in black and bold). TD (Å) stands for the interatomic distance between Valine 80 to Serine 96 or Valine 97 of TNF-α. DD (Å) stands for the interatomic distance between end residues of the replaced segment.

Nagahira K *et al* have shown that a neutralizing monoclonal antibody 3B10 binds TNF-α at position 81–88 [20]. When conjugated to KLH, the linear epitope peptide (aa 80–96) elicits weak antibody response against the native TNF-α [21] whereas the cyclic form induced stronger antibody response, indicating that a stable conformation is preferred for immunogenicity. In the crystal structure of mouse TNF-α (mTNF-α, PDB 2tnf), residues 80 to 96 form a β-hairpin structure (Fig 1A), and the interatomic distance between the end residues (aa80 and aa96) is 8.76Å.

In search for a scaffold for this epitope, we found that diphtheria toxin T-domain (DTT, aa 202–378 of DT) could be a good candidate for both conformational stabilization and adjuvanting. Firstly, the function of T-domain is engaged in translocation of the catalytic domain of DT across the endosomal membrane in low pH-dependent manner [22]. Thus there is no safety issue associated with this domain [23]. Secondly, DTT has a rigid tertiary structure composed entirely of α-helices and loops (Fig 1B). Therefore, it potentially tolerates transplantation of a foreign peptide and renders the peptide a more stable conformation [24, 25]. Moreover, DTT can be expressed in *E*.*coli* as a highly soluble protein and the CD spectrum of the purified DTT shows a typical helical conformation with two minima at 208 and 222nm (Fig 1C). This would substantially reduce manufacture costs. Thirdly, DTT possesses four T help cell epitopes (aa 69–88, 119–138, 129–148, 149–168, corresponding to DT aa 271–290, 321–340, 331–350, and 351–370), each of which is recognized by human major histocompatibility complexes (HAL) with population coverage of 88%, 71%, 82%, 70%, respectively [26]. As human MHC II genes are highly polymorphic, a combination of universal Th epitopes promises more population coverage. Among people previously immunized with DT vaccine, the Th epitopes in a DTT-based vaccine induced a rapid recall response of CD4 memory T cell [27].

As shown in Fig 1B, the DTT residues at positions 89–96 form a turn-helix-turn structure that is entirely surface-exposed. We speculated that this is a potential site for the epitope transplantation. Since the interatomic distance between the end residues of the epitope peptide is 2.88Å shorter than that of DTT aa 89 and 96, direct transplantation would potentially introduce conformational constraint to both DTT and the epitope. In order to identify appropriate transplantation that keeps the epitope conformation close to that of native molecule while DTT conformation is minimally perturbed, we generated seven epitope-scaffold molecules (Fig 1D) with difference of the interatomic distance between the epitope and the replaced segment (ΔD) varies in a range of -3.34 Å to +3.68 Å. As ΔD of DTNF3 and DTNF4 is close to each other because some of the replaced residues are at the turn of the helix, we choose DTNF4 for further experiments. DTNF7 is generated by replacing DTT 89–96 with the epitope peptide encompassing TNF-α residues 80–97. The inclusion of one more residue in the epitope peptide increases TD to 12.11 Å and reduces the ΔD to -0.47 Å.

DTNF proteins were expressed in *E*. *coli* and purified to homogeneity. All of them are highly soluble at the concentration greater than 10 mg/mL in PBS pH 7.4, 25°C. Their circular dichroism spectra are characteristic of α-helical conformation similar to that of DTT (Fig 1C).

**Fig 2:**
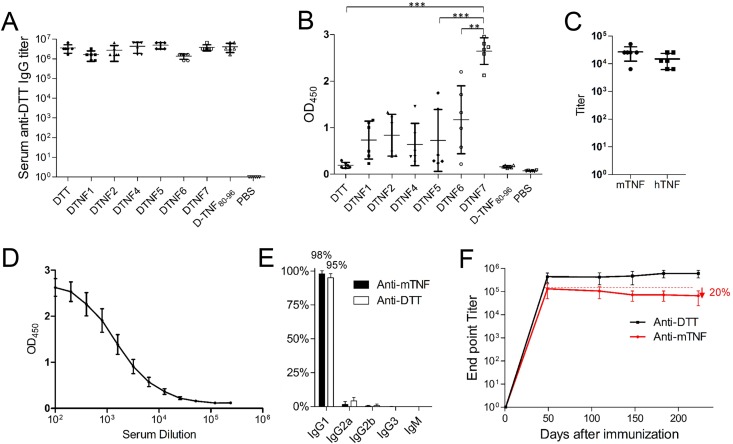
Antibody responses induced by DTT and DTNF proteins. (A) Anti-DTT antibody titers in sera of mice immunized with DTT or DTNF proteins on day 49. (B) Anti-mTNF-α antibody titers in sera of mice immunized with DTT or DTNF proteins on day 49, serum samples were diluted 1:100. (C) Serum anti-mTNF-α and anti-hTNF-α titers measured by ELISA. Serum samples were diluted 1:100. Data are presented as the mean ± SD. (D) Average anti-mTNF-α antibody titers in sera of mice immunized with DTNF7 on day 49. (E) Analysis of antibody subclass in the sera of DTNF7 immunized mice. (F) The persistent antibody response against mTNF-α and DTT in mice immunized with DTNF7. Data are presented as the mean ± SD.

Fig 2 shows that mice immunized with individual DTNF proteins generated high titers of antibody against DTT (Fig 2A) indicating that the transplantations do not interfere with the antigenicity of DTT. However, DTNF7 elicited most robust antibody response against mouse and human TNF-α, whereas the rest DTNF proteins induced much weaker responses (Fig 2B, Fig 2C and 2D), indicating that the epitope presentation is perturbed for most of the immunogens. No antibody response against native TNF-α was observed in mice immunized with DTT-peptide conjugate D-TNF_80–96_, which is consistent with a previous report showing that this peptide in linear form conjugated to KLH is a poor immunogen [14].

In DTNF7 immunized mice, over 98% of the antibodies on day 49 were IgG1 subclass (Fig 2E), which suggests that the immunoglobulin class switch has completed and the immune response is dominated by type 2 T helper cells. Remarkably, the anti-TNF-α titer was sustained for more than six months with the level decreased slightly to 80% of the maximum response on day 223 (Fig 2F), suggesting that the B cell response is mediated by activated T helper cell. In comparison with conjugate vaccines targeting TNF-α, only transient antibody responses were observed due to lack of Th-dependent B cell response [9, 10]. Our data demonstrate that the universal Th epitopes of DTT can help overcome this shortcoming. The sustained anti-TNF-α antibody response is beneficial to treatment of rheumatoid arthritis as excessive TNF-α is persistently expressed in patients.

Mice immunized with DTNF7 grew normally and their body weights, sizes of liver and spleen, and skin morphology showed no difference to control mice administered with PBS and DTT. No redness at injection sites were observed in DTNF7 immunized mice. These data implicate that the vaccine formulation is safe. Further studies is warranted to exam whether the vaccination perturb the physiological function of the cytokine and the significance thereof.

### Impact of the epitope transplantation on the conformational stability of DTT and the immunogenicity of DTNF proteins

Since ΔD values of DTNF1, 2, 5 and 6 are greater than that of DTNF7, the weaker antibody responses against TNF-α would indicate the conformational constraint caused by the transplantation affect the immunogenicity of the epitope. However, the weaker anti-TNF-α response of DTNF4 cannot be explained by the conformational constraint as ΔD value of DTNF4 is lower than that of DTNF7.

**Fig 3:**
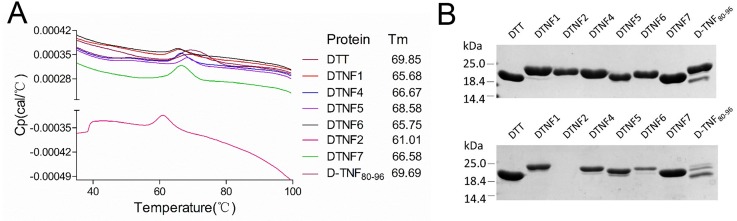
The stability of DTT and DTNF proteins. (A) Thermal stability of DTT and DTNF proteins measured by DSC. Left panel, temperature dependent heat capacity of DTT and DTNF proteins. Right panel: melting temperatures of DTT and DTNF proteins. (B) SDS-PAGE analysis of the soluble fraction of DTT and DTNF proteins stored at 4°C for 2 days (Top) and 10 days (Bottom).

It has been shown that induction of a robust antibody response is highly correlated with conformational stability of the immunogen [28–30]. Therefore, the failure of DTNF1 to 6 to induce TNF-α-specific antibody response may be due to their overall conformation stabilities. To test this possibility, we performed DSC to look into the thermal stabilities of DTNF proteins and DTT. Shown in Fig 3A, The *Tm* values of all DTNF proteins are lower than that of DTT. DTNF2 is the most unstable protein with *Tm* value ~9°C below DTT. The *Tm* values of DTNF1 and 6 are decreased by ~4°C, and those of DTNF4 and 7 decreased by ~3°C. Since DTNF4 and DTNF5 have *Tm* values slightly higher than that of DTNF7, their poor immunogenicity cannot be explained by their thermal stabilities.

**Fig 4:**
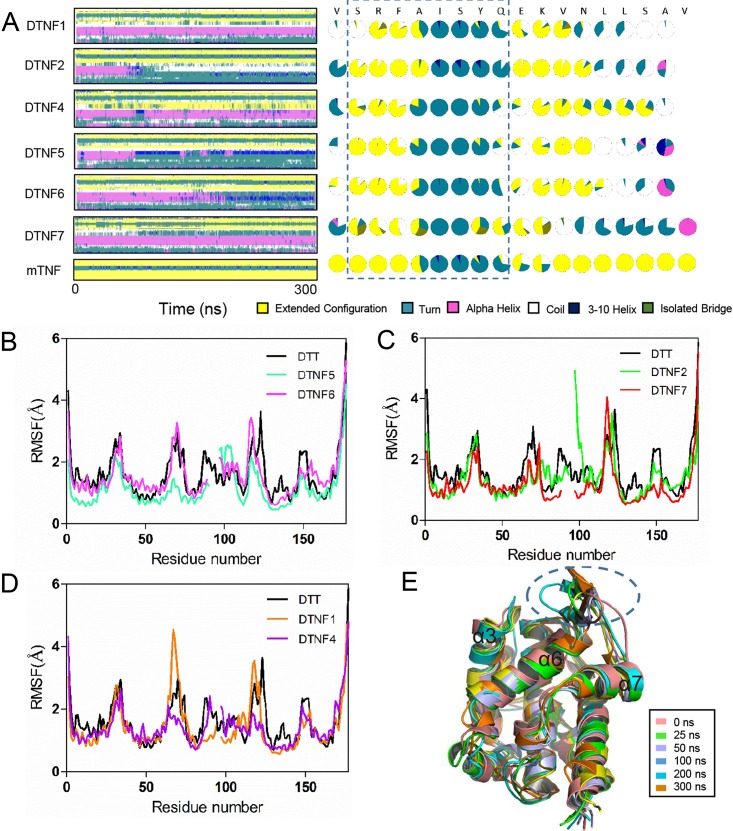
The molecular dynamics of DTNF proteins. (A) Left panel: Molecular dynamic simulation of DTNF proteins. The secondary structure conformation was shown for residues belong to TNF-α epitope and DTT at positions 97–111. Right panel: Pie charts showing fraction of time the amino acid residue adopting indicated conformation during 300 ns simulation. (B)(C)(D) The RMSF trajectories of DTT and DTNF proteins. (E) Molecular dynamic simulation of the tertiary structure of DTNF7. The dashed circle highlights the positions of TNF-α epitope peptide at indicated time.

To find out whether the stabilities of secondary structure elements could account for the immunogenicity of DTNF proteins, we performed molecular dynamics simulation. Fig 4A shows that the conformations of the TNF-α epitopes in all DTNF proteins are stable and close to that of TNF-α except DTNF1 whose Ser 81 adopts coil conformation instead of extended conformation. Shown in Fig 4E, the epitope of DTNF7 is entirely surface exposed. In contrast, conformational stability of the scaffold is perturbed to various degrees in all DTNF proteins with that of DTNF7 the least affected. The helix of DTT at position 97–101 is not stable in DTNF2, DTNF5 and DTNF6 (Fig 4B). Particularly, the helix of DTNF2 is highly flexible and its RMSF is abruptly increased compared with that of DTT (Fig 4C). The residues at position 66–69 of DTNF1 also display higher degree of flexibility. The secondary structure of DTNF4 is stable and the epitope conformation is closed to that of TNF-α. However, its RMSFs at position 123–125 are decreased compared with that of DTT, indicating a more constrained conformation of the scaffold is unfavorable for the epitope presentation (Fig 4D). Consistent with these observations, the solubility of the recombinant proteins varies after storage at 4°C for 10 days in PBS solution, and DTNF2 is especially unstable and becomes completely insoluble after the storage (Fig 3B).

### DTNF7 vaccination inhibits arthritis development in collagen-induced arthritis mouse model

**Fig 5:**
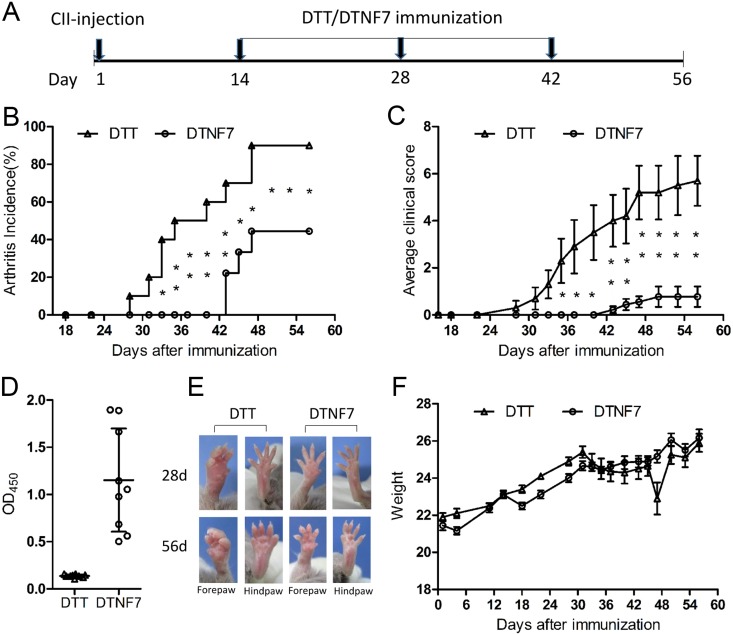
DTNF7 suppresses the development of collagen-induced arthritis in mice. (A) Mice were immunized with chicken type II collagen (CII) emulsified with CFA on day1, and immunized with alum formulated DTT or DTNF7 on day 14, 28, and 42. (B) Arthritis incidence. (C) The average clinical scores of mice immunized with DTT or DTNF7. Data are presented as the mean clinical score ± SEM (DTT, n = 10; DTNF7, n = 9). (D) The titers of anti-mTNF-α in mice sera on day 56 measured by ELISA. Sera samples were diluted 1:200. Data are presented as the mean ± SD. (E) Examples of front and hind paws on day 28 and 56. (F) Mice body weights recorded during the entire experiments.

**Fig 6:**
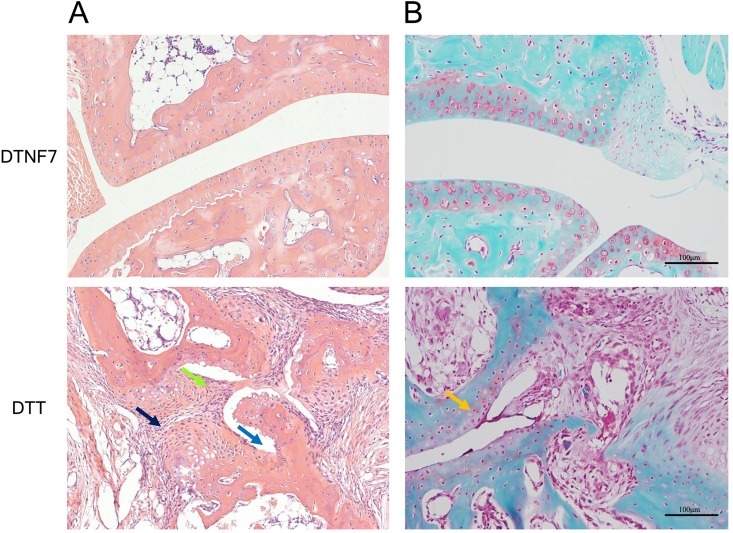
DTNF7 inhibits joint inflammation. Tissue sections of ankle joints from DTNF7 and DTT immunized mice on day 56 were stained with hematoxylin-eosin staining (A) and Safranin O staining (B). The arrows indicate synovial hyperplasia (green) cell infiltration (black), cartilage destruction (orange), and bone erosion (blue).

**Fig 7:**
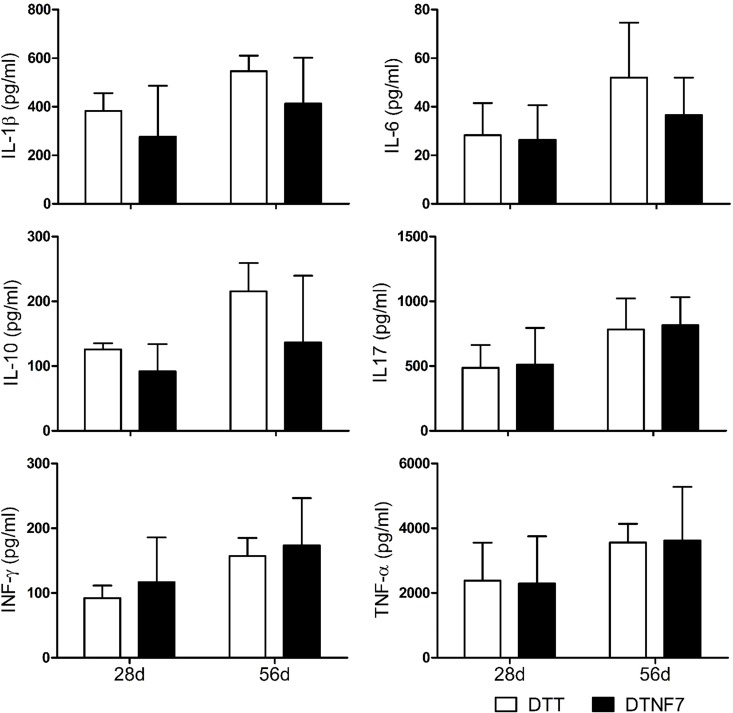
The cytokine levels in mice sera immunized with DTT or DTNF7. Serum samples were collected from CIA mice on day 28 and 56 after the first injection with chicken type II collagen. The cytokine concentrations in sera were determined by Bio-Plex assay. The average of four mice with standard variation was plotted for each cytokine. Data are presented as the mean ± SD.

To investigate the therapeutic efficacy of DTNF7, we immunized mice with the purified protein 2-weeks after collagen administration using DTT as control (Fig 5A and 5D). Compared with DTT treated mice, DTNF7 vaccination delayed the onset of arthritis by 15 days, reduced arthritis incident rate by 46% (Fig 5B), and markedly decreased the clinical arthritis score by 8 fold (Fig 5C). As shown in Fig 5E, symptoms including joint swelling and erythema were significantly improved. Consistently, histological examination of hind paws demonstrated that DTNF7 vaccination reduced synovial inflammation, cartilage destruction, bone erosion, and immune cell infiltration (Fig 6). The body weights of mice immunized with DTNF7 were similar to those immunized with DTT (Fig 5F). Moreover, the serum levels of pro-inflammatory cytokines that persist in rheumatoid arthritis including interleukin IL-1β, IL-6 and IL-10 were decreased, although the level changes had not reached statistical significance (Fig 7). Of note, the level of TNF-α was not increased after DTNF7 vaccination. This is consistent to passive immunization with a TNF-α antibody in which the levels of TNF-α is not down-regulated after administration [31].

## Discussion

Epitope peptide-based vaccines have been actively explored in the vaccine design by virtue of their targeting specificity [32, 33]. It potentially avoids allergenic and/or reactogenic effects associated with vaccines using whole organisms or large proteins [34]. However, an epitope peptide is inherently poor in immunogenicity [35], and one of the challenges in the vaccine design is to identify an appropriate and safe carrier or scaffold for structural/chemical stability and adjuvanting [36–38]. So far, no epitope-peptide vaccine has reached the commercial market yet due to lack of efficacy and/or safety concern.

In this study, we demonstrate that DTT can serve as a carrier or scaffold in vaccine design targeting self-molecules including TNF-α. We show that transplantation of a neutralizing epitope of mTNF-α into DTT at position 89–96 stabilizes the epitope in a native-like conformation while the scaffold conformation is minimally perturbed. The universal Th epitope of the DTT facilitated a T helper cell-dependent TNF-α-specific B cell activation, which is critical for a long-lasting humoral response against TNF-α [10]. Since the epitope peptide sequence is highly homologous between mouse and human, the transplantation strategy could be directly applied to human TNF-α vaccine design.

Our data also suggest that a successful vaccine design using DTT should avoid introduction of large conformational constrain to both the scaffold and peptide epitope. In addition, a stable secondary structure of the transplanted peptide is preferred as the transplanted segment of DTT is not a flexible loop. For a flexible peptide epitope, this could be achieved through engineering a disulfide bond in the epitope peptide [39, 40]. The length of the peptide should optimized to keep the span difference (ΔD) to a minimum. The solubility of the purified molecules after storage at 4°C in PBS is a good indication of its overall stability.

The B fragment of DT (DTB) is composed of T-domain and receptor binding R-domain, and has been shown an effective vaccine carrier [41]. However, DTT is better than DTB with regard to antigen load. DTT possesses only one B cell epitope whereas DTB contains several highly immunogenic B cell epitopes in the R domain [42]. The B cell epitope of DT has been shown to suppress the immune response of certain conjugated haptens by competing DT primed helper T cells [43]. In case this happen to DTT-based vaccine, appropriate mutation(s) may be introduced to modify or eliminate the B cell epitope of DTT.

Collagen-induced arthritis (CIA) is the most commonly used animal model to human rheumatoid arthritis, a chronical inflammatory autoimmune disease attacking joint. So far, TNF-α immunogens have only shown phylactic effects in CIA mouse model. DTNF7 is the first vaccine to show therapeutic effect after collagen immunization and the onset of the arthritis. The plasma level of TNF-α is extraordinarily high in the CIA model mice (2000 pg/ml) compared to that of TNF-α transgenic mice (9 pg/ml) [10]. Consequently, we were unable to determine whether the anti-TNF-α antibodies elicited by DTNF7 immunization could neutralize bioactivity with L929 assay using diluted sera. Measurements of the plasma levels of cytokines downstream of TNF-α such as IL-1β and IL-6 on day 28 and 56 were less in DTNF7 compared with DTT immunization in the CIA mice. Therefore detailed time course of more cytokine levels would reveal more insights in both the CIA model and the therapeutic mechanisms of DTNF7 immunization.

Almost all known cytokines are present in the RA synovial fluid, among which TNF-α is the first cytokine proved to be effective therapeutic target [2]. In recent years, the immune-regulatory function of GM-CSF has been shown to play crucial roles in driving the development of many autoimmune diseases [44–46]. GM-CSF or its receptor-targeted therapies have been shown highly effective and remarkably safe in treatments of many autoimmune diseases, in particular, RA [47]. Of note, antigen-specific regulatory T cells activated by therapeutics antagonizing TNF-α or GM-CSF have been shown play major immune suppressive roles in RA and other autoimmune diseases [48, 49]. This mechanism also underlies immune tolerance of ACAID (anterior chamber associated immune deviation) [50]. Studies from Shukkur M. Farooq show that collagen type II-specific ACAID Treg suppresses inflammation in CIA [51]. Therefore, it awaits further investigation to determine whether DTNF7 immunization modulate the function of collagen-specific Treg in CIA and whether the Treg cells are functionally equivalent to ACAID Treg.
